# Molecular characterization of multidrug-resistant tuberculosis against levofloxacin, moxifloxacin, bedaquiline, linezolid, clofazimine, and delamanid in southwest of China

**DOI:** 10.1186/s12879-021-06024-8

**Published:** 2021-04-08

**Authors:** Huiwen Zheng, Wencong He, Weiwei Jiao, Hui Xia, Lin Sun, Shengfen Wang, Jing Xiao, Xichao Ou, Yanlin Zhao, Adong Shen

**Affiliations:** 1 Beijing Key Laboratory of Pediatric Respiratory Infection Diseases, Key Laboratory of Major Diseases in Children, Ministry of Education, National Clinical Research Center for Respiratory Diseases, National Key Discipline of Pediatrics (Capital Medical University), Beijing Pediatric Research Institute, Beijing Children’s Hospital, Capital Medical University, National Center for Children’s Health, Beijing, China; 2 National Tuberculosis Reference Laboratory, Chinese Center for Disease Control and Prevention, Beijing, 102200 China

**Keywords:** *Mycobacterium tuberculosis*, Drug resistance, Mutation

## Abstract

**Objectives:**

To explore the drug susceptibility of levofloxacin (LFX), moxifloxacin (MFX), bedaquiline (BDQ), linezolid (LZD), clofazimine (CFZ) and delamanid (DLM) against multidrug resistant tuberculosis (MDR-TB) isolates from drug resistance survey of southwest China, and to illustrate the genetic characteristics of MDR-TB isolates with acquired drug resistance.

**Methods:**

A total of 339 strains were collected from smear-positive TB patients in the drug resistance survey of southwest China between January 2014 and December 2016. The MICs for the above mentioned drugs were determined for MDR-TB by conventional drug susceptibility testing. Genes related to drug resistance were amplified with their corresponding pairs of primers.

**Results:**

MDR was observed in 88 (26.0%; 88/339) isolates. LFX had the highest resistance rate (50.0%; 44/88), followed by MFX (38.6%; 34/88). The resistance rate to LZD, CFZ, and DLM was 4.5% (4/88), 3.4% (3/88), and 4.5% (4/88), respectively, and the lowest resistance rate was observed in BDQ (2.3%; 2/88). Of the 45 isolates resistant to LFX and MFX, the most prevalent resistance mutation was found in *gyrA* with the substitution of codon 94 (34/45, 75.6%). Two strains with CFZ - BDQ cross resistance had a mutation in the *Rv0678* gene. Of the four LZD resistant isolates, two carried mutations in *rplC* gene. For the four isolates resistant to DLM, one isolate had mutations in codon 318 of *fbiC* gene, and two isolates were with mutations in codon 81 of *ddn* gene.

**Conclusion:**

This study provided evidence of the usefulness of new anti-TB drugs in the treatment of MDR-TB in China.

## Introduction

Though effective control programmes have been implemented, TB continues to be a major lethal infectious disease worldwide, with an estimated of 10 million new cases and 1.45 million deaths in 2018 [1]. This situation is complicated further by the emergence of drug-resistant strains, dramatically limiting the treatment options. Hence, improved treatment regimens and new drugs are urgently needed to be developed. Based on the latest evidence about the balance of effectiveness to safety, the new multidrug resistant tuberculosis (MDR-TB) treatment guidelines were released in 2018 by World Health Organization (WHO) with medicines regrouped into three categories [2].

China has the second greatest number of MDR-TB cases in the world^1^. According to the Fifth National TB Epidemiological Survey, the prevalence of active and smear positive pulmonary TB in western China was greater than that in the middle and eastern regions, which was also higher than the rate of the whole country [3]. Due to the remote mountainous areas and ethnic minorities, it is more difficult to control TB in southwest China. Besides, there is a lack of resistance data on the drugs in the MDR-TB treatment regimen recommended by WHO.

The objective of this study was to explore the drug susceptibility of levofloxacin (LFX), moxifloxacin (MFX), bedaquiline (BDQ), and linezolid (LZD) from group A, clofazimine (CFZ) from group B and delamanid (DLM) from group C against MDR-TB isolates from drug resistance survey of southwest China on the basis of minimum inhibitory concentrations (MIC), and to illustrate the genetic characteristics of MDR-TB isolates with acquired drug resistance.

## Materials and methods

### Ethics statement

The protocols applied in this study were approved by the Ethics Committee of Chinese center for disease control and prevention, Beijing, China.

### Bacterial strains and culture conditions

A total of 339 strains were collected from smear-positive TB patients in the drug resistance survey of southwest of China between January 2014 and December 2016. All isolates were subcultured on the Löwenstein-Jensen (L-J) medium for 4 weeks at 37 °C.

### Conventional drug susceptibility testing

Drug susceptibility was determined using the 1% proportion method on L-J medium according to the guidelines of the WHO [4] with rifampin (RIF), 40 μg/ml; isoniazid (INH), 0.2 μg/ml; streptomycin (SM), 10 μg/ml; ethambutol (EMB), 2 μg/ml; capreomycin (CAP), 40 μg/ml; kanamycin (KAN), 30 μg/ml; and ofloxacin (OFX), 2 μg/ml. The MDR-TB was defined as resistance to at least INH and RIF, and XDR-TB was defined as any MDR strain additionally resistant to one fluoroquinolone and one second-line injectable drug.

### Minimum inhibitory concentrations

For MDR-TB identified by conventional drug susceptibility testing, the MICs of LFX, MFX, LZD, BDQ, CFZ, and DLM were determined as described previously [5]. The MIC value was defined as the lowest concentration of antibiotic that inhibits visible growth of mycobacteria. By referring to previous literature [6–8], susceptibility breakpoints for each antibiotic are shown in Table 1. *Mycobacterium tuberculosis* H37Rv (ATCC 27249) was used as the control strain.

**Table 1:** The minimum inhibitory concentrations and susceptibility breakpoints for each antibiotic

Drug	MIC range (mg/l)	No. of isolates in the MIC											Breakpoint (mg/l)	No. (%) of resistant strains
		≤0.015	0.03	0.06	0.12	0.25	0.5	1	2	4	8	16		
LFX	0.12–8	0	0	0	18	24	2	10	10	20	4	0	0.5	44 (50.0)
MXF	0.06–4	0	0	20	16	7	11	9	15	10	0	0	0.5	34 (38.6)
BDQ	0.015–2	67	10	5	2	2	1	1	0	0	0	0	0.25	2 (2.3)
LZD	0.03–2	0	9	7	35	27	5	1	4	0	0	0	1	4 (4.5)
CFZ	0.06–4	0	0	59	17	3	6	0	2	1	0	0	1	3 (3.4)
DLM	0.015–1	83	1	0	0	0	0	4	0	0	0	0	0.125	4 (4.5)

### DNA extraction and sequencing

Genomic DNA was extracted from freshly cultured bacteria as previously reported [9]. Genes related to drug resistance were amplified with their corresponding pairs of primers (Table 2). The 25 μl PCR mixture was prepared as follows: 12 μl of 2× Taq Master Mix, 1 μl of forward and reverse primers (10 μΜ), 10 μl of distilled H_2_O and 1 μl of genomic DNA. PCR parameters for amplification were 5 min at 95 °C, followed by 35 cycles at 94 °C for 1 min, 58 °C for 1 min, 72 °C for 1 min, and a final extension at 72 °C for 10 min. PCR products were sent to Tsingke company for sequencing. Sequencing data was aligned with the corresponding sequences of the *M. tuberculosis* H_37_Rv reference strain using Bioedit (version 7.1.3.0) software.

**Table 2:** Primers used in this study for amplification and sequencing

ATB drug	Gene	Primer		Length
FQs	*gyrA*	Forward	TGACATCGAGCAGGAGATGC	320 bp
		Reverse	GGGCTTCGGTGTACCTCATC	
	*gyrB*	Forward	GTGGAAATATGTTGGCCGTC	413 bp
		Reverse	GTCGTTGTGAACAACGCTGTG	
LZD	*23S rRNA*	Forward	GGTTGAAGACTGAGGGGATGAG	2422 bp
		Reverse	CATCGGCGCTGGCAGGCTTAG	
	*rplC*	Forward	GCTGCGGCTGGACGACTC	420 bp
		Reverse	CTCTTGCGCAGCCATCACTTC	
	*rplD*	Forward	CCGGGCGGATGGGCAATGACC	936 bp
		Reverse	GGAATCCGGGCGCACCAAAAAC	
BDQ	*atpE*	Forward	TGTACTTCAGCCAAGCGATGG	454 bp
		Reverse	CCGTTGGGAATGAGGAAGTTG	
	*pepQ*	Forward	ATCAATGCCCCCTGGAAC	1371 bp
		Reverse	GCACGTTCTTCAACTTGGTG	
CFZ	*rv1979c*	Forward	GCGGCGGAAATGAGTGT	1647 bp
		Reverse	ATGCACGACGGCTTTATCA	
BDQ/ CFZ	*rv0678*	Forward	TGCCTTCGGAACCAAAGAA	795 bp
		Reverse	GACAACACGGTCACCTACAA	
DLM	*fbiA*	Forward	CGGTTCTGTTGTGGTTGGG	1136 bp
		Reverse	CCGATGACGGGCAGGATC	
	*fbiB*	Forward	GCCGCTGCTGATGACCGA	1494 bp
		Reverse	TCGGGAGGTTGATGGTTGG	
	*fbiC*	Forward	GTCCACCGCTCTGCCGAGTC	2386 bp
		Reverse	GCCACCTTCGAGCATCACC	
	*fgd1*	Forward	TCGCGTTTATGGCATAGGAGT	1090 bp
		Reverse	ACTTACCCGTCTGCGATTCTG	
	*ddn*	Forward	CACCATCATCGAGCGGATTT	765 bp
		Reverse	CAAGGGCGTGAAATGGGAT	

### Statistical analysis

SPSS v.17.0 (SPSS Inc., Chicago, IL) was used to carry out *χ*^2^ analysis, and differences were considered to be statistically significant if *P* < 0.05.

## Results

### Drug susceptibility profiles of MDR-TB strains

MDR was observed in 88 (26.0%; 88/339) isolates with 59 (67.0%; 59/88) resistant to SM, 28 (31.8%; 28/88) resistant to EMB, 6 (6.8%; 6/88) resistant to CAP, 11 (12.5%; 11/88) resistant to KAN, and 35 (39.8%; 35/88) resistant to OFX. In addition, 9 isolates (10.2%; 9/88) were identified as XDR strains.

The results of MICs for MDR-TB isolates against six antibiotics were shown in Table 1. LFX had the highest resistance rate (50.0%; 44/88), followed by MFX (38.6%; 34/88). The resistance rate to LZD, CFZ, and DLM was 4.5% (4/88), 3.4% (3/88), and 4.5% (4/88), respectively, and the lowest resistance rate was observed in BDQ (2.3%; 2/88). In addition, out of the 44 LFX-resistant isolates, 33 (75%) were resistant to MFX, and 2 (66.7%) out of the 3 CFZ-resistant isolates were resistant to BDQ.

### Mutations conferring antibiotics resistance

Among the 45 isolates resistant to FQs (44 isolates resistant to LFX, 34 isolates resistant to MFX, and 33 isolates cross resistant to LFX and MFX), the most prevalent resistance mutation of *gyrA* was the substitution in codon 94 (34/45, 75.6%), resulting in the amino acid substitution of Asp for 13 (13/45, 28.9%) Gly, 11 (11/45, 24.4%) Tyr, 6 (6/45, 13.3%) Ala, and 4 (4/45, 8.9%) Asn. In addition, the second most common substitution occurred in codon 90 with substitution of Ala-Val (8/45, 17.8%). The remaining substitutions were Gly88Cys (1/45, 2.2%), Asp89Asn (1/45, 2.2%), Ser91Pro (1/45, 2.2%). Notably, substitution Ser95Thr in *gyrA* known to be not associated with FQs resistance was found in all FQs resistance isolates. No nonsynonymous mutations were identified in the *gyrB* gene (Table 3).

**Table 3:** Mutations conferring antibiotices resistance

Gene	Nucleotide Substitution	Amino acid change	ATB drug	Number of isolates
*gyrA*	G262T	Gly88Cys	LFX	1
			MXF	1
	G265A	Asp89Asn	LFX	1
			MXF	1
	C269T	Ala90Val	LFX	8
			MXF	6
	T271C	Ser91Pro	LFX	1
			MXF	1
	G280T	Asp94Tyr	LFX	11
			MXF	11
	G280A	Asp94Asn	LFX	4
			MXF	4
	A281C	Asp94Ala	LFX	6
			MXF	5
	A281G	Asp94Gly	LFX	13
			MXF	9
	G284C	Ser95Thr	LFX	44
			MXF	34
*Rv0678*	A152G	Gln31Arg	BDQ	1
			CFZ	1
	T157C	Ser53Pro	BDQ	1
			CFZ	1
*rplC*	T460C	Cys154Arg	LZD	2
*ddn*	G241A	Gly81Ser	DMD	2
*fbiC*	G952A	Val318Ile	DMD	1

A total of 3 strains were identified as CFZ resistant, sequence analysis revealed that all these strains harbored no nucleotide substitution in the *rv1979c* gene. And no mutation was observed in *atpE* gene of BDQ resistant isolates. All two CFZ-BDQ cross resistant strains had a mutation in the *Rv0678* gene, one with Gln31Arg substitution and the other with Ser53Pro.

Of the four isolates resistant to LZD, two isolates carried mutations in *rplC* gene with amino acid substitution of Cys154Arg, while *23S rRNA* and *rplD* gene seemed not to confer LZD resistance among these strains.

In addition, five candidate genes associated with DLM resistance was also analyzed in four DLM-resistant isolates. No mutations were observed in *fbiA*, *fbiB*, and *fgd1* genes, while one isolate with the mutations in codon 318 of *fbiC* gene and two isolates in codon 81 of *ddn* gene.

## Discussion

Since the MDR-TB cases have emerged as a major obstacle to global TB control due to small number of effective drugs and high risk of adverse effects, promising candidates are needed to provide potential solutions to resolve this troublesome dilemma. Following a clinical study of novel regimens recommended for MDR-TB, we investigated drugs resistance from the groups A, B and C, and to analyze the genetic determinants of this resistance in MDR-TB isolates obtained from patients.

For the two newer FQs, our data revealed that 50.0% of MDR-TB strains were resistant to LFX, and 38.6% resistant to MFX in southwest of China. A study from mid-East of China showed that 37% of fluoroquinolone resistance events were in MDR *M. tuberculosis* isolates [10]. And a total of 76% LFX resistance and 73% MFX resistance MTB isolates were identified in a study from southern China [11]. Another report from Shanghai revealed that among FQ-resistant *M. tuberculosis* strains, 44% were multidrug-resistant isolates [12]. The higher resistance rate of FQs in China may due to the overuse of FQs in the treatment of undiagnosed bacterial infections, highlights the urgent need to take action to fight FQs misuse in clinical practice. Molecular analysis showed that a total of 75.6% FQr isolates were shown to harbour mutations in *gyrA* QRDR, with condon 94 being the most predominant mutation site, but no mutation in *gyrB* gene was observed [12]. The reported frequency in condon 94 is similar to that in Hong Kong (75%) [13] and to that in Rwanda (75%) [14], although it is lower than that in Russia (83%) [15] and higher than that in Taiwan (50%) [16], showing that mutations in *gyrA* QRDR were the key factor leading to quinolone resistance in *M. tuberculosis* in China.

As for BDQ and CFZ, more than 95% of MDR-TB strains were susceptible in this study. Though they are now being studied as a component of novel regimens for MDR-TB, mutations in the *Rv0678* gene, which causes the overexpression of efflux pump MmpS5-MmpL5, were found to result in cross-resistance between the two drugs [17]. Consistent with previous studies, our data demonstrated that two of three CFZ-resistant isolates had *Rv0678* mutations and were cross-resistance to BDQ. However, no mutations in *Rv1979c* were identified in CFZ-resistant isolates without *Rv0678* mutations. Further studies are needed to expand our understanding of mechanisms of resistance to CFZ by identifying additional mutations. Though several target-based resistance mutations in the *atpE* gene have been described in BDQ-resistant strains, but no *atpE* mutations were found in this study. Additional mechanisms may be contributed to the resistance of BDQ.

Linezolid was effectively used to treat of MDR strains [6]. According to previous studies, the T460C mutation in *rplC* gene was the most frequent among the LZD-resistant isolates [18]. In this study, two MDR-TB isolates (2/4, 50%) were identified mutations in the *rplC* gene, which was in line with previous studies. In addition, polymorphisms in any of five genes (*ddn, fgd1, fbiA, fbiB,* and *fbiC*) have been shown to lead to in vitro DLM resistance [8]. *Ddn* gene, encoding an F_420_-dependent mycobacterial nitroreductase, was associated with the prodrug activation. Resistance to DLM is caused by the loss of this ability to activate DLM [19]. In a recent study by Liu et al. [20], out of the ten primary resistant isolates, five were MDR-TB isolates with nonsynonymous mutation on *ddn*. In this study, two MDR isolates with *ddn* mutation conferring DLM resistance were identified. Besides, previous studies revealed that mutations in the coenzyme F_420_ biosynthesis pathway result in the loss of *M. tuberculosis* bacilli’s ability to activate PA-824, another nitroimidazole similar to DLM [21]. So as a member of the coenzyme F_420_ biosynthesis pathway, *FbiC* was involved in DLM resistance. In this study, of the three DLM resistant isolates, one isolate with mutation in *fbiC* gene, suggesting *fbiC* serves as an important contributor to DLM resistance.

## Conclusions

In conclusion, MDR-TB isolates exhibited a high proportion of resistance to FQs, whereas excellent activity against MDR-TB was observed in BDQ, LZD, DLM and CFZ in southwest of China. Strains with mutations in codon 94 of the *gyrA* gene are more likely to be associated with high-level FQs resistance. In addition, mutations in *Rv0678* gene and *rplC* gene were related to clofazimine-bedaquiline cross resistance and linezolid resistance respectively, and mutations in *fbiC* and *ddn* gene may be conferred to delamanid resistance on *M. tuberculosis* isolates. And this study provides evidence for further application of new anti-TB drugs in the treatment of MDR-TB in China.

## Data Availability

Data supporting the results can be found in this paper. The datasets generated during and analyzed during the current study are available from the corresponding author shenadong16@hotmail.com (AD. Shen) on reasonable request.
